# The potential effect of instrumentation with different nickel titanium rotary systems on dentinal crack formation—An *in vitro* study

**DOI:** 10.1371/journal.pone.0238790

**Published:** 2020-09-09

**Authors:** Márk Fráter, András Jakab, Gábor Braunitzer, Zsolt Tóth, Katalin Nagy

**Affiliations:** 1 Department of Operative and Esthetic Dentistry, Faculty of Dentistry, University of Szeged, Szeged, Hungary; 2 dicomLAB Dental Ltd., Szeged, Hungary; 3 Department of Oral Biology and Experimental Dental Research, Faculty of Dentistry, University of Szeged, Szeged, Hungary; 4 Department of Oral Surgery, Faculty of Dentistry, University of Szeged, Szeged, Hungary; Danube Private University, AUSTRIA

## Abstract

The potential mechanical impact of different rotary systems used for root canal preparation has been a matter of debate for long. The aim of this study was to explore the incidence of dentinal cracks after root canal instrumentation with various rotary systems, *in vitro*. One hundred and eighty intact lower central incisors were selected and randomly divided into fourteen treatment groups (n = 12/group) and a control group (n = 12). After decoronation, the root canals were instrumented with fourteen different rotary systems (E3, E3 azure, NT2, Hyflex CM, Hyflex EDM, 2Shape, OneCurve, ProTaper Next, ProTaper Gold, WaveOne Gold, Mtwo, Reciproc Blue, TF adaptive, K3XF). All roots were horizontally sectioned at 3, 6, and 9 mm from the apex with a low-speed saw under water-cooling. The slices were then examined under stereomicroscope for dentinal cracks. No cracks were found in the control group. Cracks were found in all treatment groups, predominantly in the 3 mm slices. There was no statistically significant difference in the number of cracks when comparing the different systems to each other at any section level. At 3 mm, however, five of the studied systems, namely K3XF (p = 0.004), Protaper Next (p = 0.001), Reciproc Blue (p<0.001), TF adaptive (p = 0.050), and 2Shape (p = 0.009) presented a significantly higher number of cracks than the control group. Within the limitations of this study, instrumented canals presented dentinal cracks, while uninstrumented ones presented no cracks after sectioning. There seems to be no significant difference among the tested systems regarding crack formation in the instrumented root canal wall. Crack formation occurred irrespective of the motion of the rotary system (rotational or reciprocation). Further studies are needed to clarify the factors that contribute to crack formation in the case of each individual rotary system.

## Introduction

Root canal debridement is an umbrella term used to describe various mechanical instrumentation procedures aimed at removing inflamed and/or infected pulp tissue and microbial biofilm attached to the dentin. Furthermore the aim of such procedures is to prepare the root canal for filling [1]. The high flexibility and shape memory of nickel titanium (NiTi) instruments has made them the first choice for root canal debridement [2]. They are easier to use, allow faster preparation [3], and decrease the risk of canal transportation due to their flexibility [4]. Despite these undeniably favorable features, even the latest NiTi instruments suffer from a number of characteristic issues. Of those, unexpected fracture may be the most frequently reported one [5–7]. Furthermore, the greater dentin contact with these files during root canal preparation results in momentary stress accumulation, which has been suggested to induce craze line and crack formation in the wall of the canal [2, 8].

There is ongoing interest in assessing crack formation by the different instruments used for root canal preparation. The question is typically studied either by microscopic evaluation after sectioning or CT evaluation. So far the results are controversial. De-Deus et al. [9–12] and PradeepKumar et al. [13] showed that root canal preparation does not cause dentinal cracks when evaluated by CT scans, whereas the results of Aksoy et al. [14] and Bayram et al. [15] indicate just the opposite. In general, microscopic studies report a higher number of cracks than CT scans, which raises the possibility that sectioning itself distorts the results. The fact that multiple studies failed to find dentinal cracks in sectioned control teeth [16–21] contradicts that argument.

Recently, manufacturers have developed NiTi superelastic alloys (e.g.: R-phase, M-wire, C-wire, CM-wire, T-wire, Gold-wire) with special thermomechanical processing. HyFlex CM and HyFlex EDM systems (Coltene/Whaledent AG, Altstatten, Switzerland) are both manufactured from controlled memory (CM) wire, which provides significant fatigue resistance, ease of bending, and the ability to return to its original shape when heated above the transformation temperature [22, 23]. Protaper Gold files (Dentsply Maillefer, Ballaigues, Switzerland) are manufactured from Gold-wire, which results in increased flexibility [15]. Protaper Next files (Dentsply Maillefer) contain M-wire and incorporates a variable taper design, giving the file an eccentric rotational motion [24]. The K3XF and TF Adaptive systems (Sybron Endo, Orange, CA) contain an intermediate phase (R-phase) between martensite and austernite. While the K3XF files perform rotational movement, the TF Adaptive instrument can change from continuous rotation to reciprocation in order to reduce the stress during preparation [18]. The Mtwo system (VDW, Munich, Germany), the E3 files (Endostar, Poldent Co. LTD, Warsaw, Poland) and the NT2 files (Endostar) are made of conventional (untreated) NiTi, while the E3 azure (Endostar) files are exposed to a specially designed heat-treatment process. Not only does the latter result in superior flexibility, but the file can also be pre-bent before insertion into the canal. Both the OneCurve system and 2Shape system (Micro-Mega, Besancon, Cedex, France) are manufactured from superelastic NiTi alloys (C-wire and T-wire).

Single-file systems have been developed to shape the canal with only a single file and reduce the time required for the preparation process. The previously characterized HyFlex EDM system and the OneCurve system are single-file rotary systems. Reciproc Blue (VDW, Munich, Germany) and WaveOne Gold (Dentsply Maillefer) represent the commercially available single-file reciprocating systems. During the fabrication of Reciproc Blue, an innovative heating process is utilized to modify the molecular structure of the material to improve its flexibility and resistance to cyclic fatigue [14].

While a considerable number of studies have investigated the ability of various endodontic instrumentation systems to create cracks, no study has thus far undertaken a comprehensive cross-system analysis in this respect. Therefore, this *in vitro* study was designed to investigate the formation of dentinal microcracks after root canal preparation with multiple NiTi systems applying a sectioning approach with microscopic analysis. The null hypotheses were that there is no quantitative difference in the formation of dentinal microcracks (1) between the tested groups (including control) at the same section levels, and (2) between different section levels within the same rotary system.

## Materials and methods

All procedures of the study were approved by the Ethics Committee of the University of Szeged, and the study was designed in accordance with the Declaration of Helsinki (reference number of the granted permission from the Human Investigation review Board: 4029). It is mandatory to receive patient’s informed consent and permission for tooth extraction, therefore this is always received at the time of extraction as part of the routine documentation. Also patients are informed that the extracted teeth could be used for training or research purposes, and both verbal and written approval (by signature) is obtained from the patients at this point. Human mandibular central incisors with mature apices, extracted for periodontal reasons were selected and stored in distilled water for not more than 3 weeks. Radiographs were taken, and only single-rooted teeth with a single straight canal (<5° inclination) were included in the study. The teeth were standardized according to their mesiodistal (3.5–4 mm) and buccolingual (5.5–6.5 mm) width. All the roots were inspected with a stereomicroscope (Carl Zeiss Technival, Carl Zeiss, Germany) at 40× magnification to detect any pre-existing external defects or cracks. Teeth with such defects and those outside the standard dimensions were excluded from the study. Following these preliminary selection procedures, 180 teeth were selected for the study.

### Root canal preparation

The coronal portions of all teeth were removed using a low-speed saw (Isomet 1000; Buehler, Lake Bluff, IL) under water cooling, leaving roots approximately 13 mm in length. In the majority of the teeth, the canal width near the apex was compatible with a size 10 K-file (Dentsply Maillefer). Otherwise, the tooth was excluded from the study and replaced by one that fit that criterion. The working length was established by subtracting 1 mm from the length of a size 10 K-file inserted into the canal until the tip of the file became visible at the apical foramen. In addition, to model real-life conditions as close as possible, the surface of the roots were coated with a latex separating agent (Rubber-Sep, Kerr, Orange, CA) in order to simulate the elasticity of periodontal ligaments and then embedded into methacrylate resin (Technovit 4004, Heraeus-Kulzer, Hanau, Germany). The specimens were randomly allocated to one control group (Group 1) and 14 treatment groups (Groups 2 to 15). Treatment groups were defined by the instrument used for preparation. All groups contained 12 specimens. Shaping procedures were performed with 14 different NiTi rotary systems as follows: Group 2: Endostar E3, Group 3: Endostar E3 azure, Group 4: Endostar NT2, Group 5: Hyflex CM, Group 6: Hyflex EDM, Group 7: 2Shape, Group 8: OneCurve, Group 9: ProTaper Next, Group 10: ProTaper Gold, Group 11: WaveOne Gold, Group 12: Mtwo, Group 13: Reciproc Blue, Group 14: TF adaptive, Group 15: K3XF (see Table 1 for details.) All systems were used according to the manufacturers’ instructions. If resistance necessitating more apical pressure was felt, the file was removed and the flutes were cleaned. This was repeated for each file until reaching the working length. To standardize the apical enlargement, the canals were instrumented to an apical preparation size of 25, and the taper of the files was 0.06 or as close to 0.06 as possible within each system. Instrumentation sequences for each system are given in Table 1. In each group, the full sequence recommended by the manufacturer, irrespective of the number of the files, was used before reaching the designated apical enlargement to avoid any undesirable strain of each file. After each instrument insertion, the teeth were irrigated with 2 ml 3% sodium hypochlorite. After completion of the procedure, canals were rinsed with 2 ml distilled water. The same expert operator performed all root canal preparations, and 2 blinded operators checked the presence of dentinal defects or lack thereof.

**Table 1:** Details and characteristics of rotary instruments used for root canal instrumentation.

Name of system	Taper (%)	MAF	Sequence (Size/Taper)	Type of movement
VDW Mtwo	6	25	10/4% -> 15/5% -> 20/6% -> **25/6%**	Rotary
Kerr K3™ XF	6	25	25/8% (preflaring) -> 40/6% -> 35/6% -> 30/6% -> **25/6%**	Rotary
Dentsply Sirona ProTaper Next	Variable	25	16/2% -> 17/4% -> **25/variable**	Rotary
Micro-Mega One Curve	6	25	25/9% (preflaring) -> 25/3% (glide-path) -> **25/6%**	Rotary
VDW Reciproc Blue	Variable	25	**25/variable**	Reciprocating
Coltene HyFlex™ CM	6	25	25/8% (preflaring) -> 20/4% -> 25/4% -> 20/6% -> **25/6%**	Rotary
Kerr TF™ adaptive	6	25	20/4% -> **25/6%**	TF™ adaptive
Micro-Mega 2Shape	6	25	25/9% (preflaring) -> 25/3% (glide-path) -> 25/4% -> **25/6%**	Rotary
Coltene HyFlex™ EDM	Variable	25	10/5% (glide-path) -> **25/variable**	Rotary
Dentsply Sirona ProTaper Gold	Variable	25	16/2% -> 18/2% -> 20/4% -> 20/7% -> **25/variable**	Rotary
Endostar E3	6	25	20/6% -> 25/4% -> 20/4% -> 25/4% -> **25/6%**	Rotary
Endostar E3 azure	6	25	20/6% -> 25/4% -> 20/4% -> 25/4% -> **25/6%**	Rotary
Endostar NT2	2	25	15/2% -> 20/2% -> **25/2%**	Rotary
Dentsply Sirona WaveOne Gold	7	25	15/2% (glide-path) -> **25/7%**	Reciprocating

MAF, Master apical file.

### Microscopic evaluation

All roots were horizontally sectioned at 3, 6, and 9 mm from the apex with a low-speed saw (Isomet 1000; Buehler, Lake Bluff, IL) under copious water cooling. The slices were then inspected both with a dental operating microscope (Carl Zeiss Omni Pico, Oberkochen, Germany) and the stereomicroscope under illumination. Dentin sections were illuminated with a technique described by Coelho et al [25]. Light-emitting diode (LED) transillumination was performed with the aid of a probe (TransCure-T; Kinetic Instruments Corporation, Bethel, CT) and within 1 mm of the external surface of the root. Samples containing cracks were photographed with a reflex camera (Nikon D90; Nikon Tokyo, Japan) attached to the dental operating microscope.

**Fig 1:**
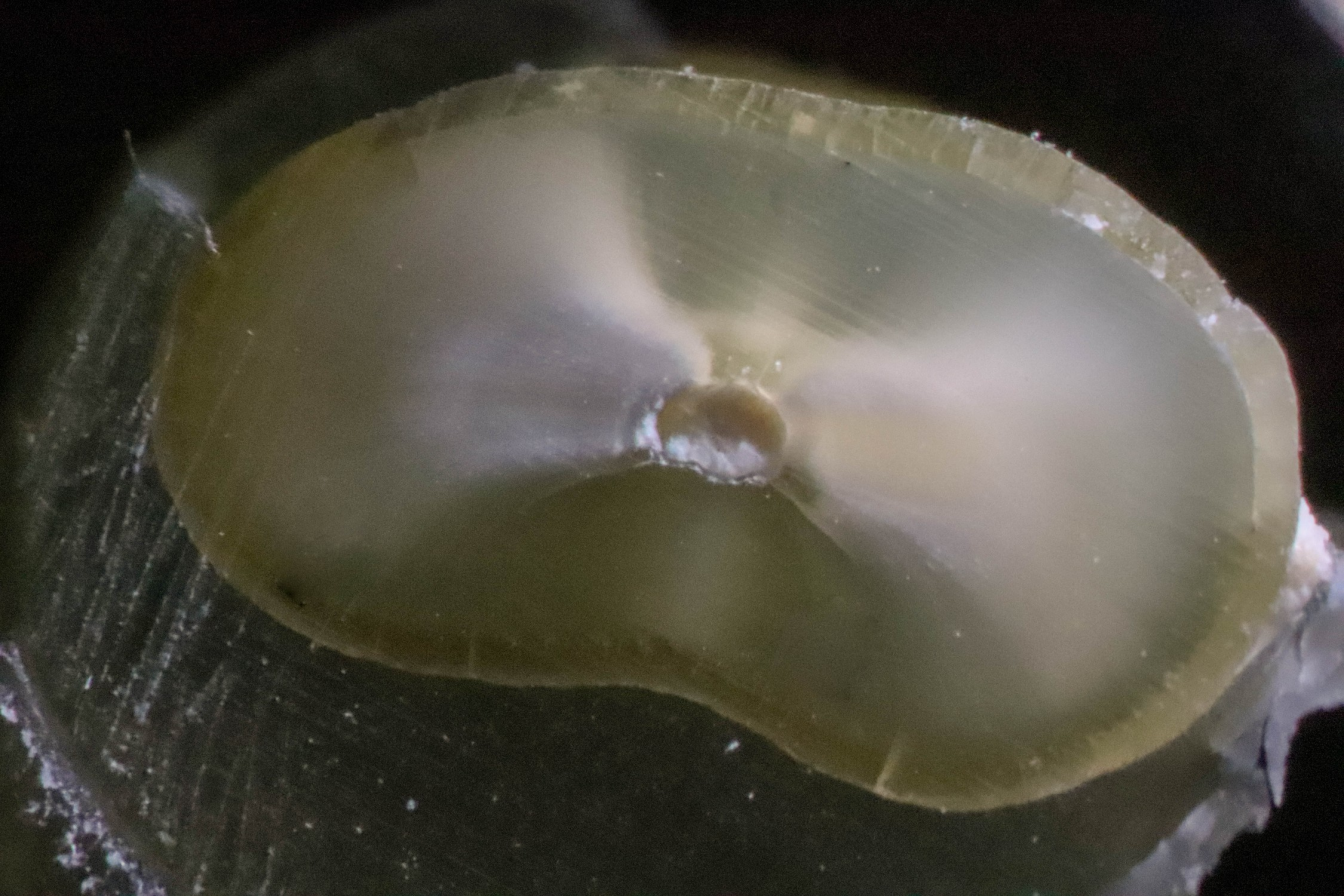
Cross section of an instrumented specimen with a visible microcrack.

**Fig 2:**
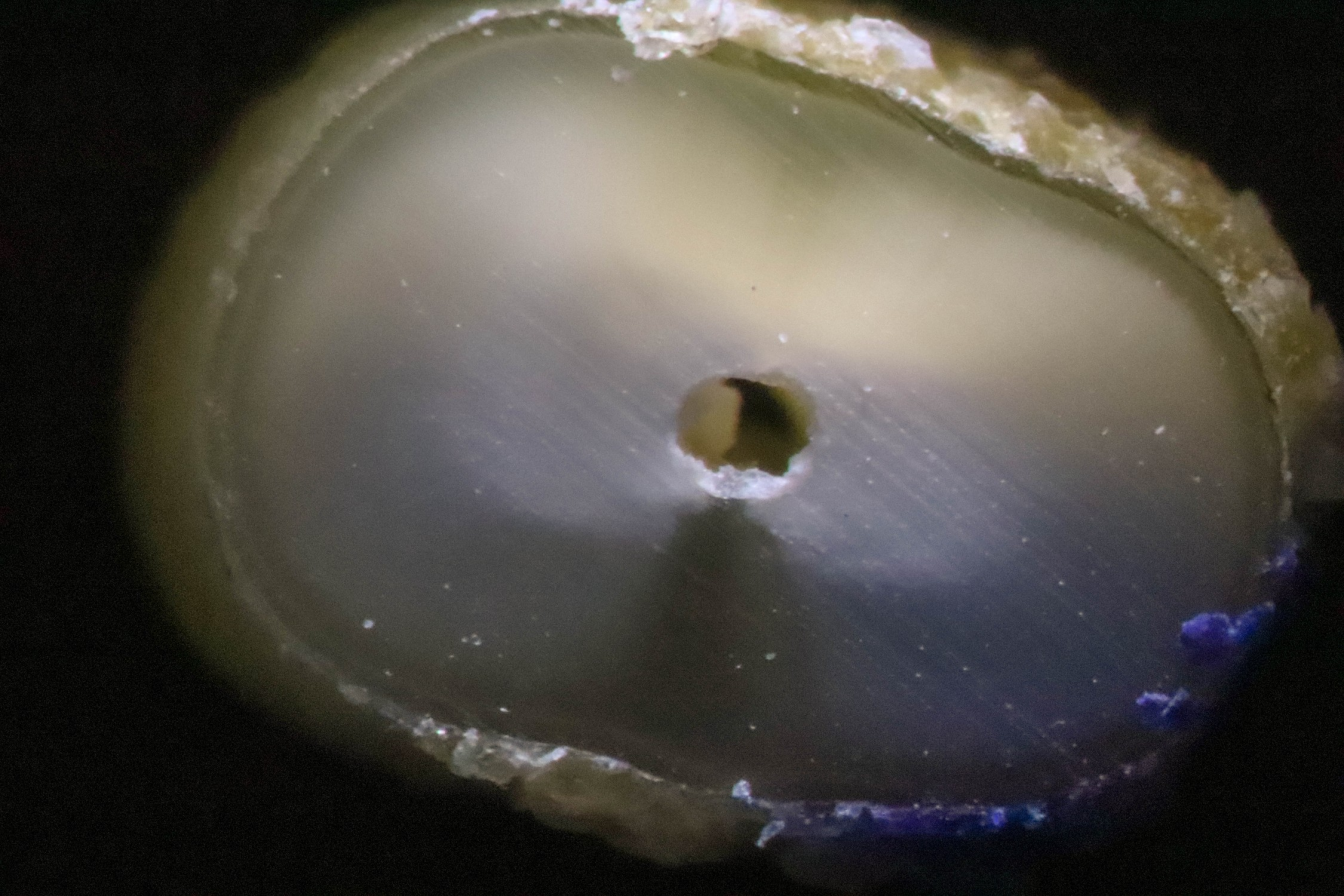
Cross section of a control specimen (unprepared tooth) without any crack.

A crack was defined according to Li et al. as originating from the inner root canal space [16] (Fig 1). Other defects that did not originate from the canal wall, such as craze lines, were not considered cracks (Fig 2). Roots were classified as cracked if at least 1 of the 3 sections obtained from each root showed at least 1 crack [16, 26]. In total, 180 teeth were available for evaluation, resulting in a total of 540 sections to be viewed and evaluated by 2 calibrated and blinded observers.

### Statistical analysis

Statistical analysis was conducted in SPSS 23.0 (SPSS, Chicago, IL). For the comparisons between the groups, ANOVA with Tukey's HSD post-hoc test was used. The general limit of significance was set at α = 0.05 (corrected for the multiple comparisons).

## Results

**Fig 3:**
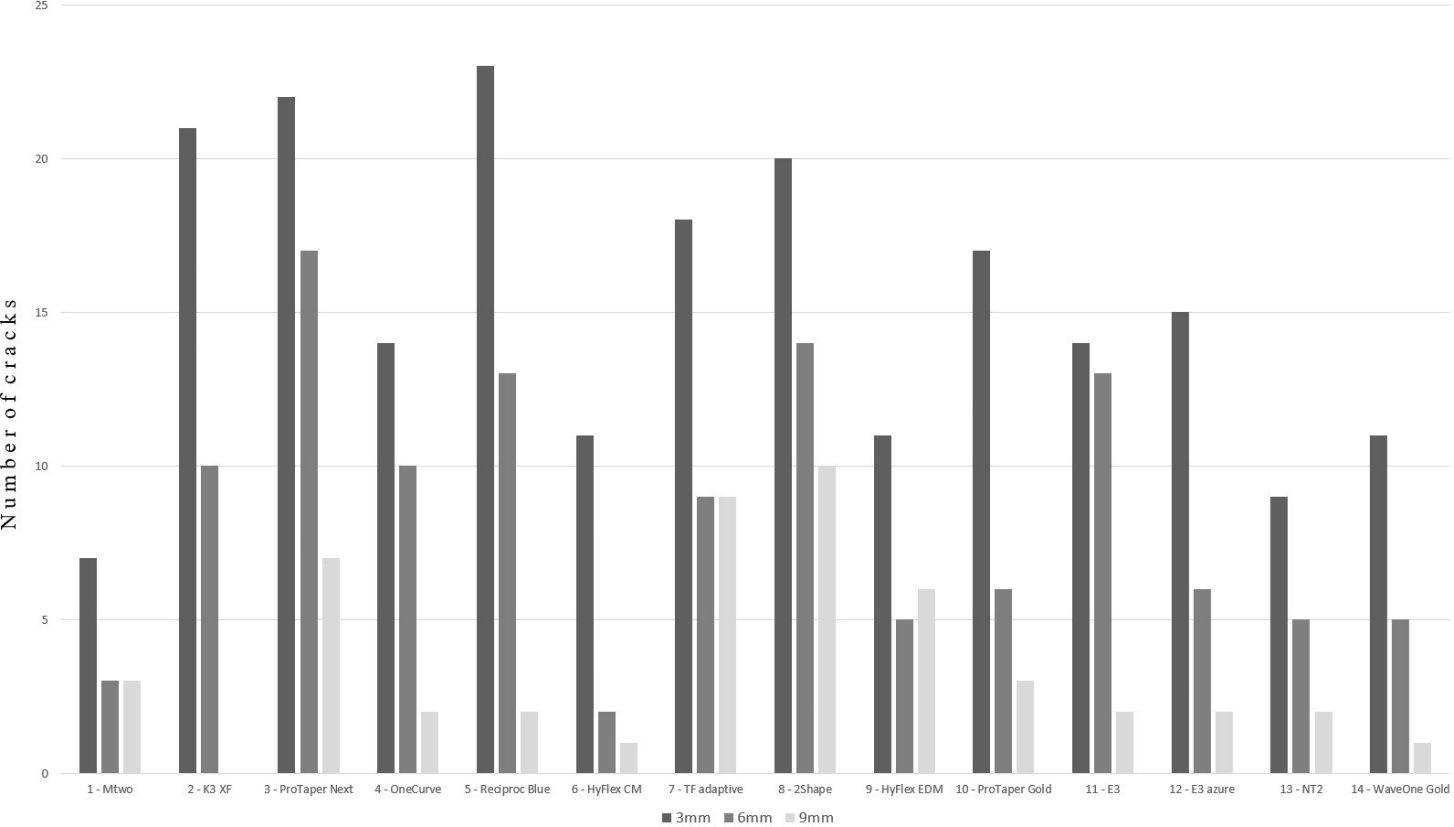
Number of cracks in the different cross sections.

Fig 3 summarizes the number of cracks identified at different section levels for the different treatment groups. No cracks were observed in the control group, whereas all tested rotary systems caused cracking. When comparing the samples at 9 mm for all groups (including control), there was no statistically significant difference in the number of cracks. The same applied for all the samples at 6 mm. However, when comparing the 3 mm section levels for all groups, five of the studied systems (K3XF, p = 0.004; Protaper Next, p = 0.001; Reciproc Blue, p<0.001; TF adaptive, p = 0.050; 2Shape, p = 0.009) caused a significantly higher number of cracks in the root canal treated samples than in the control group. Therefore, the first null hypothesis was rejected.

The frequency of cracking increased in all tested groups in the corono-apical direction. There was no statistically significant difference in the number of cracks when comparing the samples at 9 mm and at 6 mm, or comparing at 6 mm and at 3 mm within the same system. Interestingly, when comparing the number of cracks at 3 mm and at 9 mm section within the same system, there were two systems (K3XF and Reciproc Blue) that generated a statistically significantly higher number of cracks at the more apical level (p = 0.004; and p = 0.004, respectively). Thus, the second null hypothesis was rejected.

## Discussion

There is a multitude of NiTi systems on the market, and dentists face difficulty when trying to find studies comparing most of them in a clinically relevant way. This *in vitro* study tried to address this problem by comparing the microcrack formation by multiple NiTi instrumentation systems in extracted teeth. We included instruments with different kinematics and, for the first time, both multiple-file and single-file systems were included in the analysis. Mandibular incisors were used because they are highly susceptible to fracture due to their narrow mesiodistal dimension [27].

When comparing the number of cracks at different root levels, there was no significant difference between the different systems at 9 or 6 mm from the apex. This is in accordance with the findings of Karataş et al. [18], but not with the findings of Pedulla et al. where the number of cracks differed significantly across single-file systems at 6 mm and 9 mm [19]. Also, the number of cracks present at 6 mm and 9 mm did not differ statistically from the control group at the same level. It seems from our findings that irrespective of the motion of the file system (rotational or reciprocating), the design of the file, or the presence or absence of pre-flaring, dentinal crack formation due to instrumentation cannot be deemed a significant issue in the coronal or middle part of the canal.

Although the number of cracks increased apically (see Fig 3), the difference was not significant within the same rotary system between the 3 mm and 6 mm levels. Also, the within-system comparisons indicated no significant difference between the 3 mm and the 9 mm levels, except for K3XF (p = 0.004) and Reciproc Blue (p = 0.004). In these systems, we found significantly more cracks at the 3 mm (apical) section than at 9 mm. The reason for this is probably that the apical part is the narrowest part of the canal, therefore any instrument contacts with the greatest canal surface in this part. Interestingly, this difference was not so pronounced in case of the other systems. Within the K3XF system multiple files with mainly 6% conicity are used in a crown-down technique with a rotational movement. Contrary to the mentioned system, Reciproc Blue is a single-file system working with a reciprocating motion. Despite being manufactured from a blue superelastic alloy which provides significant flexibility, Reciproc Blue has been documented to produce dentinal cracks during instrumentation [28].

When comparing the apical 3 mm sections across the instrumented groups, there was no statistically significant difference. However, significant differences were found between five systems (K3XF, p = 0.004; Reciproc Blue, p<0.001; TF adaptive, p = 0.050; 2Shape, p = 0.009; and Protaper Next, p = 0.001) and the control group. This is in line with other studies showing that all tested single-file systems produced significantly more cracks at the apical 3 mm compared to intact teeth [19]. However, only Reciproc Blue is a single-file system among the above mentioned 5 systems. Reciproc Blue, Protaper Next and 2Shape files are manufactured with a special NiTi alloy (M‑wire) subjected to a special thermal treatment intended to increase the flexibility of the instrument [29]. TF adaptive is produced in a manufacturing process that involves heat treatment (R-phase), twisting of the metal wire, and special surface conditioning [30]. As a unique feature it combines rotational and reciprocating movement. K3XF was developed with the same R-phase heating and cooling protocol as TF adaptive, but instead of being twisted, it is ground [31].

Regarding the kinematics of file systems, reciprocating movement is safer both with respect to cyclic fatigue and torsion fracture [32]. As a result, the life span of the instruments used with this motion is longer [33]. According to a meta-analysis, reciprocating files generate significantly fewer cracks than conventional multiple-file rotary systems with pure rotational movement (e.g. Protaper, Mtwo, etc.) [34]. Our findings suggest that this claim may need to be refined, since this in our study differed according to the level of sectioning (apical, middle or coronal). Furthermore, neither Reciproc Blue, nor WaveOne Gold (both performing reciprocating movement) differed significantly in the number of cracks when compared to the other tested systems. Thus, kinematics does not appear to be the sole factor to influence crack formation. Previous studies found that TF adaptive produced significantly fewer cracks than fully reciprocating systems (Reciproc and WaveOne) [29, 18]. In contrast, we found that TF adaptive did not differ significantly from either Reciproc Blue or WaveOne Gold in such terms.

In summary, it is safe to assume that crack formation cannot be traced back to a single factor. Instead, it is a result of multiple, possibly additive and/or synergistic factors, such as tip design, cross-sectional design, taper, manufacturing process of the NiTi alloy, etc. [35].

Regarding the limitations of this study, it must be clarified that the *in vitro* approach could introduce false positives because of the extraction forces the specimens had been exposed to when they were harvested, the conditions of storage, and the sectioning procedure [25, 36]. Sectioning procedure hold an uncertainty as if it is not accompanied by CT scan already existing dentinal cracks cannot be ruled out. Also the sectioning procedure itself might contribute to the formation of the dentinal crack, however, the chance that it could significantly influence the results is extremely low as non-treated (but sectioned) control specimens exhibited no cracks at all. Several other studies came to the same conclusion [16–21]. The problems regarding the utilization of extracted teeth has been emphasized by De-Deus et al. [37], however, current findings seem to contradict the possible role of extraction in dentinal crack formation inside the root canal [38]. All in all, while, theoretically, it is impossible to rule out the influence of these factors, the complete lack of cracks in the control specimens is a strong argument against the role of the above mentioned factors within this study setup.

As there is still no clear consensus on the interpretation of the results gathered from microscopic evaluations after sectioning and CT evaluations, future research should be done with both evaluation methods in the same samples. The need for future research in this aspect has been clearly emphasized by Zaslansky et al. [39]. Furthermore, our results are can be considered valid for an apical preparation size of 25. Future studies should address larger apical preparation also.

As for the strengths of this study, the tested systems were used according to the manufacturer’s instructions (i.e. no modification or simplification was made for the purposes of the study), which makes the study clinically relevant. While such simplifications are allowable for purely experimental purposes, the manufacturer’s recommendations should never be disregarded in a clinical setting. Furthermore, when choosing a system for clinical practice, the potential effect of the full sequence should be evaluated.

## Conclusions

Within the limitations of this *in vitro* study, root canal preparation in straight root canals with an apical preparation size of 25 does influence dentinal crack formation inside the root canal. Certain rotary systems seem to be more aggressive in terms dentinal crack development compared to others. Dentinal crack development cannot be traced back to a single factor (e.g.: kinematics of the file, number of files in the sequence, presence or absence of pre-flaring)., it is probably multi-factorial instead.

## Supporting information

S1 File Significance matrix from the post-hoc pairwise comparisons (Tukey’s HSD). Empty cells indicate lack of significance. Numbers in specific cells show p-values indicating significant differences. C: control (unprepared teeth). Group 2: Endostar E3, Group 3: Endostar E3 azure, Group 4: Endostar NT2, Group 5: Hyflex CM, Group 6: Hyflex EDM, Group 7: 2Shape, Group 8: OneCurve, Group 9: ProTaper Next, Group 10: ProTaper Gold, Group 11: WaveOne Gold, Group 12: Mtwo, Group 13: Reciproc Blue, Group 14: TF adaptive, Group 15: K3XF, (3): section 3 mm from the apex, (6): section 6 mm from the apex, (9): section 3 mm from the apex.
(XLSX)
